# Monitoring data on the effect of domestic livestock and rabbits on *Androcymbiumeuropaeum* (Lange) K. Richt. and its xerophytiques pastures for thirteen years

**DOI:** 10.3897/BDJ.12.e113943

**Published:** 2024-02-26

**Authors:** Antonio Jesús Pérez-Luque, María Eugenia Ramos-Font, Mauro José Tognetti Barbieri, Clara Montoya Román, Claudia Tribaldos Anda, Francisco M. Cabezas-Arcas, José Luis González-Rebollar, Ana Belén Robles

**Affiliations:** 1 Estación Experimental del Zaidín-Consejo Superior de Investigaciones Científicas, Granada, Spain Estación Experimental del Zaidín-Consejo Superior de Investigaciones Científicas Granada Spain; 2 Institute of Forest Sciences ICIFOR, INIA-CSIC. Ctra. La Coruña km 7.5, 28040, Madrid, Spain Institute of Forest Sciences ICIFOR, INIA-CSIC. Ctra. La Coruña km 7.5, 28040 Madrid Spain

**Keywords:** occurrence, endangered plant species, Ibero-Maghreb endemism, herbivory, species richness, species diversity, Cabo de Gata-Níjar Natural Park (southern Spain), plant conservation

## Abstract

**Background:**

Dataset of annual monitoring of herbivory effects on the conservation status of the endangered species *Androcymbiumeuropaeum* (Lange) K. Richt and its associated plant communities is presented in this manuscript. This dataset encompasses the annual monitoring of herbivory effects on the conservation status of the endangered species *Androcymbiumeuropaeum*. Since 2010, the SERPAM Department (Service of Evaluation, Restoration and Protection of Mediterranean Agrosystems) at the Zaidin Experimental Station, belonging to the Spanish National Research Council (CSIC-EEZ), has conducted annual sampling to assess the impact of both domestic and wild livestock, specifically rabbits, on the pastures where *A.europaeum* lives. The study consisted of a randomised block design, implementing three distinct treatments to evaluate different management strategies: (1) rabbit and domestic herbivory, (2) exclusion of domestic livestock and (3) exclusion of rabbits and domestic livestock. Within each treatment, two types of monitoring were conducted. Firstly, the abundance of *A.europaeum* was estimated by counting individuals within 50 cm x 50 cm quadrats. Secondly, plant species diversity was assessed along 2-m long transects using the modified Point-Quadrat method. The research was conducted within the Cabo de Gata-Níjar Natural Park in southern Spain, specifically in the Amoladeras Nature Reserve in Almería.

**New information:**

The dataset contains information spanning from 2010 to 2023, providing valuable insights into the annual monitoring of herbivory effects on the conservation status of *A.europaeum*, contributing to our understanding of the species' interaction with domestic and wild animal in the studied area.

## Introduction

*Androcymbiumeuropaeum* (Lange) K. Richt., commonly known as Cape saffron or "Azafrán del Cabo" (in Spanish), is an endemic plant found exclusively in the Ibero-Maghreb Region. Belonging to the Colchicaceae family, this species is a winter-growing geophyte that undergoes vegetative development from October to March, strongly dependent of rainfall patterns. The flowering period of this plant occurs during autumn and winter, displaying a strong correlation with precipitation levels and temperature variation (Robles et al. 2014). Each plant produces 2-3 (occasionally 6) flowers, with pollination being facilitated by beetles, flies and bees. Remarkably, the pollen fertility rate is exceptionally high, at 99%. Following the onset of flowering, the first fruits emerge within 15-20 days and, under favourable conditions, almost all flowers bear fruit. As the aerial parts begin to desiccate from March onwards, the mature fruits detach and their seeds are dispersed after about a year (Caujape-Castells and Pedrola-Monfort 1997). Germination of these seeds is facilitated by lower temperatures.

*Androcymbiumeuropaeum* inhabits the thermo-Mediterranean belt characterised by a semi-arid to arid ombroclimate, specifically, in xerophytic grasslands and open clearings, often found on stony or sandy substrates. The soil typically consists of skeletal, stony or sandy terrain, occasionally revealing limestone bedrock outcrops. This species is commonly found within winter ephemeral therophytic grasslands, which exhibit varying coverage and are rich in plant species (Alcaraz et al. 1989, Moreno Saiz et al. 2019, Monserrat 1961). It is typically part of the edge of extensive scrublands dominated by jujube (*Ziziphuslotus*) vegetation, a priority habitat within the Habitats Directive (Directive 92/43/EEC) (Blanca et al. 1999, Tirado 2009).

**Figure 1.**
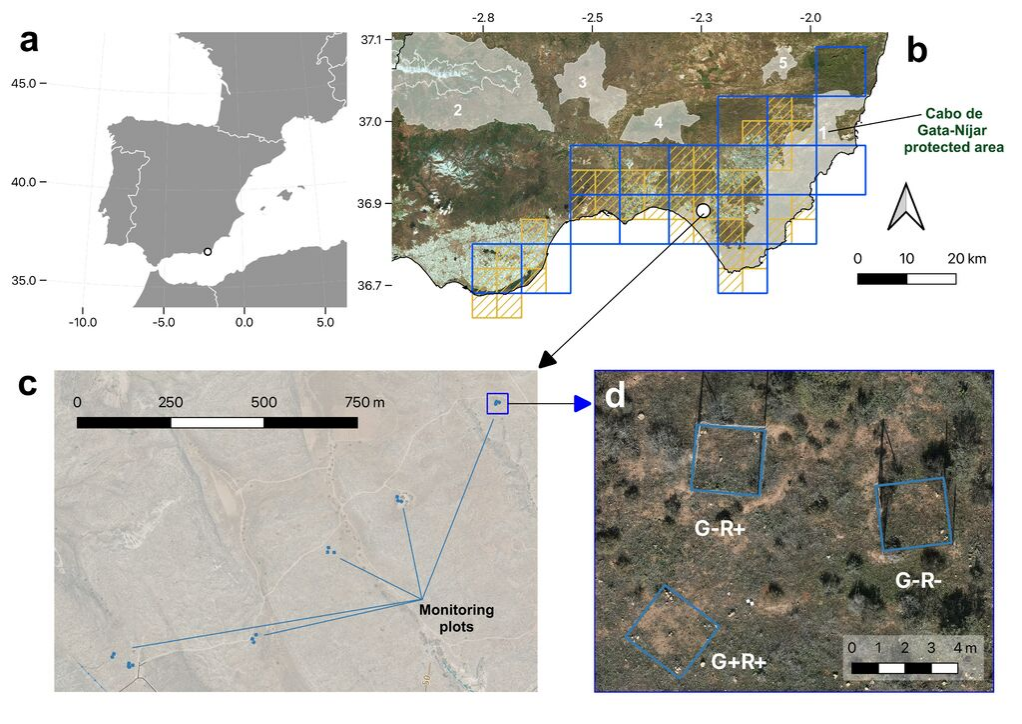
Distribution and location of the study plots**. a** Location of the study area; **b** Distribution of the 10 km x 10 km (*blue squares*) and 5 km x 5 km (*yellow squares*) grids in which *A.europaeum* has been cited at national (Moreno Saiz et al. 2019) and regional level (Consejería de Agricultura, Ganadería, Pesca y Desarrollo Sostenible. Junta de Andalucía 2022). Shaded polygons indicate natural protected areas (1: Cabo de Gata-Níjar Natural Park; 2: Sierra Nevada National and Natural Park; 3: Desierto de Tabernas, 4: Sierra Alhamilla and 5: Karst en Yesos de Sorbas Natural sites). The white circle indicates the study area; **c** Detailed map with the location of the monitoring plots in Amoladeras, Almería; **d** High detailed orthophotography view of one the monitoring blocks with the three different treatments: herbivory by sheep and rabbits (G+R+); excluding only sheep (fenced with hunting netting) (G-R+); and excluding rabbits and sheep (G-R-).

Regarding its distribution, *A.europaeum* is primarily found in the south-eastern region of the Iberian Peninsula, in the Province of Almería (Fig. 1a), as well as in western Morocco. Currently, five populations are known, comprising between 255,000 and 630,000 individuals, scattered over 17 10 km x 10 km grid squares (Fig. 1b), with an area of occupancy distributed over 133 1 km x 1 km grid (Moreno Saiz et al. 2019). The population sizes of *A.europaeum* undergo notable yearly fluctuations, primarily influenced by the ever-changing climatic conditions. Moreover, in Almeria, its area of distribution has been reduced and it is classified as vulnerable. Since 1994, it has been included in the Catalogue of Threatened Wild Flora of Andalusia and, in 2000, it was included in the Red List of the Spanish Vascular Flora (Moreno 2008). The transformation of land use, characterised by the establishment of greenhouses of vegetables, extensive road construction, the development of tourist facilities and the relentless expansion of urban areas, plays a pivotal role in the deterioration of the species' habitat. In addition, other factors such us overgrazing, abandonment of agricultural land, mining operations, improper waste disposal, as well as the introduction and uncontrolled spread of alien and invasive plant species, are threatening the populations of this species, as it occurs with other threatened species located on the coast of Almería (Mendoza-Fernández et al. 2015).

Several species within the Cochicaceae family have exhibited a positive relationship between the presence of small herbivores and livestock and their population density (Borghi and Giannoni 1997, Gomez-García et al. 2009). Interestingly, some species within this family are consumed by herbivores despite containing highly toxic alkaloids (Gómez-García et al. 2003). *A.europaeum*, which shares similar secondary metabolites, such as colchicine, demelcocine, desmethylcolchicine and colchifolin, has been found to possess these compounds in both its vegetative and reproductive organs (Osem et al. 2002, Ellington et al. 2003), but this species vary across different locations (Boza et al. 1998). Additionally, the creeping growth habit of *A.europaeum*, with its leaves positioned close to the ground, suggests an adaptation to herbivory (Esler et al. 1999, Noy‐Meir and Oron 2001). The south-eastern region of the Iberian Peninsula, where *A.europaeum* is found, is known for its notable rabbit population and the persistence of traditional extensive grazing practices involving sheep and goats (Verdu and Galante 2002, Alados et al. 2004). As a result, rabbits, sheep and goats can be regarded as potential consumers of *A.europaeum*.

To enhance our comprehension of grazing's impact on the population dynamics of *A.europaeum*, we have been conducting a comprehensive monitoring programme at a specific site for the past 13 years. Our objective is to evaluate the contribution of both domesticated and wild animals in the status conservation of this species, while also examining the diversity of the pastures that serve as habitats for *A.europaeum*. This long-term study aims to deepen our understanding of the interplay between grazing practices and the conservation of this species. This data-paper has focused on documenting a dataset to assess the impact of domestic livestock and rabbits on the density of *A.europaeum* (in excluded to livestock and rabbits and non-excluded plots) over a period of thirteen years (2010-2023) in one of the most well-preserved populations of the Cabo de Gata-Níjar Natural Park.

## Project description

### Title

Monitoring data on the effect of domestic livestock and rabbits on *Androcymbiumeuropaeum* pastures

### Personnel

Ana Belén Robles Cruz (Principal Investigator); José Luis González Rebollar, María Eugenia Ramos-Font, Mauro José Tognetti Barbieri, Antonio Jesús Pérez-Luque, Francisco Mario Cabezas-Arcas, Clara Montoya Román, Claudia Tribaldos Anda.

### Design description

**Figure 2.**
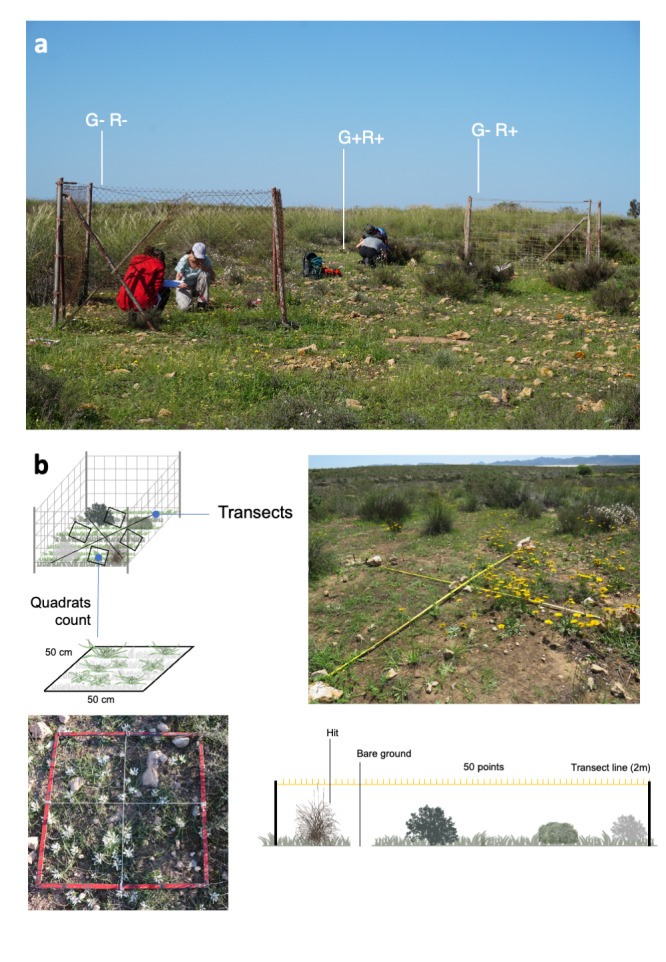
Monitoring scheme showing the three treatments per block (**a**) and detail of the transects and the quadrats (**b**).

To evaluate the effect of sheep and rabbits on the population of *A.europaeum* and its plant communities, 18 plots of 2.5 m x 2.5 m were installed in the study area (Fig. 2). A randomised block design was followed and consisted of six blocks separated between 300 and 400 m, with three different treatments or management types (one plot by treatment and block): **1)** with herbivory by sheep and rabbits (G+R+), **2)** excluding only sheep (G-R+) (fenced with hunting netting) and **3)** excluding rabbits and sheep (G-R-) (fenced with rhomboidal netting with a 4 cm mesh). Within each plot, the abundance of *A.europaeum* in each plot and year was evaluated by counting the number of individuals in 50 cm x 50 cm fixed squares, taking four quadrats per plot, distributed according to the four cardinal points (N, S, E and W): 24 quadrats per treatment (six blocks by four quadrats). The exclusion plots were installed in May 2010, after the first density sampling (March); thus, this year should be considered as year zero, without exclusion treatments. The density of *A.europaeum* was assessed between January and March, depending on the species phenology. Within each above-mentioned plot, two 2-m fixed-crossed transects were set to assess plant community composition, species richness, diversity and cover.

### Funding

These data have been generated thanks to the funding of different projects, although the first samplings and the installation of the exclusion plots were funded by the Consejería de Medio Ambiente de la Junta de Andalucía through the "*Ganadería Extensiva y Biodiversidad*" project from 2008 to 2014. From this date on, it was financed through the CSIC intramural projects "*Investigaciones sobre flora forrajera bética: prospección de especies, protocolo para su establecimiento en campo y valoración nutritiva*" and "*Pastoralismo y Medioambiente*". From 2021 onwards, funding for monitoring came from the SUMHAL project (*Sustainability for Mediterranean Hotspots in Andalusia integrating LifeWatch ERIC)* (LIFEWATCH-2019-09-CSIC-04, POPE 2014-2020).

## Sampling methods

### Sampling description

To evaluate the effect of sheep and rabbits on the population of *A.europaeum* and its plant communities diversity, 18 plots of 2.5 m x 2.5 m were installed in the study area. A randomised block design was followed, which consisted of six blocks separated between 300 and 400 m (Fig. 1c), with three different treatments or management types (one plot by treatment and block) (Fig. 1d): 1) with herbivory by sheep and rabbits (G+C+); 2) excluding only sheep (G-C+) (fenced with hunting netting) and 3) excluding rabbits and sheep (G-C-) (fenced with rhomboidal netting with a 4 cm mesh) (Fig. 2a).

Abundance of *Androcymbiumeuropaeum*

The density of *A.europaeum* in each plot was yearly evaluated by counting the number of individuals in 50 cm x 50 cm fixed squares, taking four quadrats per plot, distributed according to the four cardinal points (N, S, E and W): 24 quadrats per treatment (six blocks by four quadrats) (Fig. 2b). As mentioned before, the exclusion plots were established in May 2010, following the initial density and biodiversity samplings (March and April).; thus, this year should be considered as year zero, without exclusion treatments.

Assessment of plant communities

The evaluation of plant communities where *A.europaeum* lives was conducted annually during the spring season to ensure that most annual species had grown and flowered, allowing for accurate identification. In each plot, the point-intercept (non-destructive) method was applied, following a modification proposed by Daget and Poissonet (1971) of the point-quadrat method originally described by Levy and Madden (1933). Two fixed crossed transects measuring 2 m in length were established (Fig. 2b), with 50 points surveyed per transect. These points were spaced 4 cm apart and the plant species in contact with a 2 mm needle were recorded. For each transect, the following variables were determined:

- Coverages:
  - specific plant cover: percentage of soil covered by each plant species
  - vegetation cover: percentage of soil covered by vegetation
  - total specific plant cover: sum of the specific vegetation cover of each species
  - musk/lichen cover: percentage of soil covered by musk or lichen
  - bare soil cover: percentage of bare soil
- Floristic composition:
  - plant species richness: number of species
  - Shannon diversity index (H'): \begin{varwidth}\begin{equation*}
            \text{H'} = -\sum p_i \cdot \ln(p_i)
        \end{equation*}\end{varwidth} where \begin{varwidth}\begin{equation*}
            p_i
        \end{equation*}\end{varwidth} is the proportion of individuals of one particular species found divided by the total number of individuals found.

### Quality control

For the abundance count and to reduce possible bias amongst observers, criteria for the correct identification of individuals were established and practised prior to sampling for each field campaign. In general, the results obtained from the beginning were quite coincident and without inter-observer bias.

Each year, sampling was carried out by 2-4 observers, with at least one of them present in all the samplings. Plants for almost all species found were sampled and determined in the lab. Data where carefully implemented in a database and cross-check validations were carried out.

The sampling plots were georeferenced using a Kolida K20S high-precission GPS with an accuracy of ± 10 mm. Digital colour orthophotographs derived from flights performed with an RGB camera (45 Megapixels; Zenmuse P1, DJI) on board a UAV drone (DJI Matrice 300 RTK) were also used to verify that the geographic coordinates of each sample plot were correct. The spatial data were originally recorded as UTM using the datum EPSG:25830, but were transformed to geographic coordinates (EPSG: 4326) for easy manipulation.

The specimens were taxonomically identified using Flora Vascular de Andalucía Oriental (Blanca et al. 2011), Flora Ibérica (Castroviejo 2021) and Flora Europaea (Tutin et al. 1980). The scientific names were checked with GBIF Backbone Taxonomy (GBIF Secretariat 2022). We also used the R package *taxize* (Chamberlain and Szocs 2013) to verify the taxonomical classification.

The data were accommodated to fulfil the Darwin Core Standard (Wieczorek et al. 2012, Darwin Core Maintenance Group 2021a, Darwin Core Maintenance Group 2021b). We used Darwin Core Archive Validator tool (http://tools.gbif.org/dwca-validator/) to check whether the dataset met Darwin Core specifications. The Integrated Publishing Toolkit (Robertson et al. 2014) of the Spanish node of the Global Biodiversity Information Facility (GBIF) (http://www.gbif.es/ipt) was used both to upload the Darwin Core Archive and to fill out the metadata.

### Step description

All data were stored in a normalised database using Microsot Access. Custom-made SQL views of the database were performed to gather event and occurrence data. In addition, some variables at transect level were computed (see Sampling description section). Data were exported and taxonomic and spatial validations were made on this database (see Quality-control description).

Structure of the Darwin Core Archive (DwC-A).

The DwC-A encompasses comprehensive sampling event data (GBIF 2018) consisting of *event-type* data, *occurrence* data and *extended measurement or fact* type data. This structure is organised, based on a hierarchical framework for sampling events. The event file contains a total of 1583 records distributed as follows: 18 events that describe the spatial coverage of the plots (with three plots per block and six blocks), 72 events that detail the spatial coverage of each of the four quadrat counts within each plot and 36 events that outline the spatial coverage of the two transects within each plot. The events related to quadrat count and transects contain *aparentEventID* field, linking them to their respective plots. A total of 1456 records correspond to the temporal visits made to each of the aforementioned events (Fig. 4). The occurrence file comprises 4011 records, while the "extended measurement or fact" file encompasses 6922 records of various measurements associated with the transect and quadrat events.

In order to facilitate the users with utilisation of the dataset, we wrote a detailed tutorial of how to download, process and prepare data from the GBIF dataset. This tutorial is available at https://ajpelu.github.io/dp_androcymbium_lab/ (Pérez-Luque 2023).

## Geographic coverage

### Description

The study area belongs to the Integral Reserve of Las Marinas-Amoladeras, one of the main steppe zones of the Cabo de Gata-Níjar Natural Park, located in Almería (southern Spain) (Fig. 1). This area is considered a hunting refuge and zoological reserve. The limestone soils are poor and poorly developed. Biogeographically, it belongs to the Murcian-Almerian chorological province, Almeria sector, thermo-Mediterranean semi-arid-arid belt. The annual precipitation is 200 mm with strong intra- and interannual variation and the mean annual temperature is 19ºC. The potential vegetation of the area consists of *Ziziphuslotus* thornscrub, but at present, shrubs of the *Helianthemo-Siderition pusillae* alliance together with *Eryngio ilicifoli-Plantaginetum ovatae* and *Androcymbio-Tillaetum muscosae* alliances dominate (Rivas-Martínez et al. 2002), reflecting the former grazing-pastoral activity, now almost abandoned and of which only occasional pastoral use with sheep remains, together with hunting activity.

### Coordinates

36.831783 and 36.8381261 Latitude; -2.2435419 and -2.2552436 Longitude.

## Taxonomic coverage

### Description

*A.europaeum* has undergone different nomenclatural changes throughout its history, having been assigned to the genera *Melianthium* L., *Erythrostictus* Schltdl. or *Androcymbium* Willd. (Bellot Rodríguez 1946). Its most widespread name is *A.gramineum*; in fact, it is accepted in several regional, national and even European lists; however, recent molecular studies have concluded that *Androcymbium* is a paraphyletic group of the genus *Colchicum* L. (Manning et al. 2007); thus, the currently accepted name of *A.europaeum* is *Colchicumeuropaeum* (Lange) J.C. Manning & Vinnersten. In this work, we use the synonym *A.europaeum* because it is widely accepted and used in most of the taxonomic lists and floras consulted.

**Figure 3.**
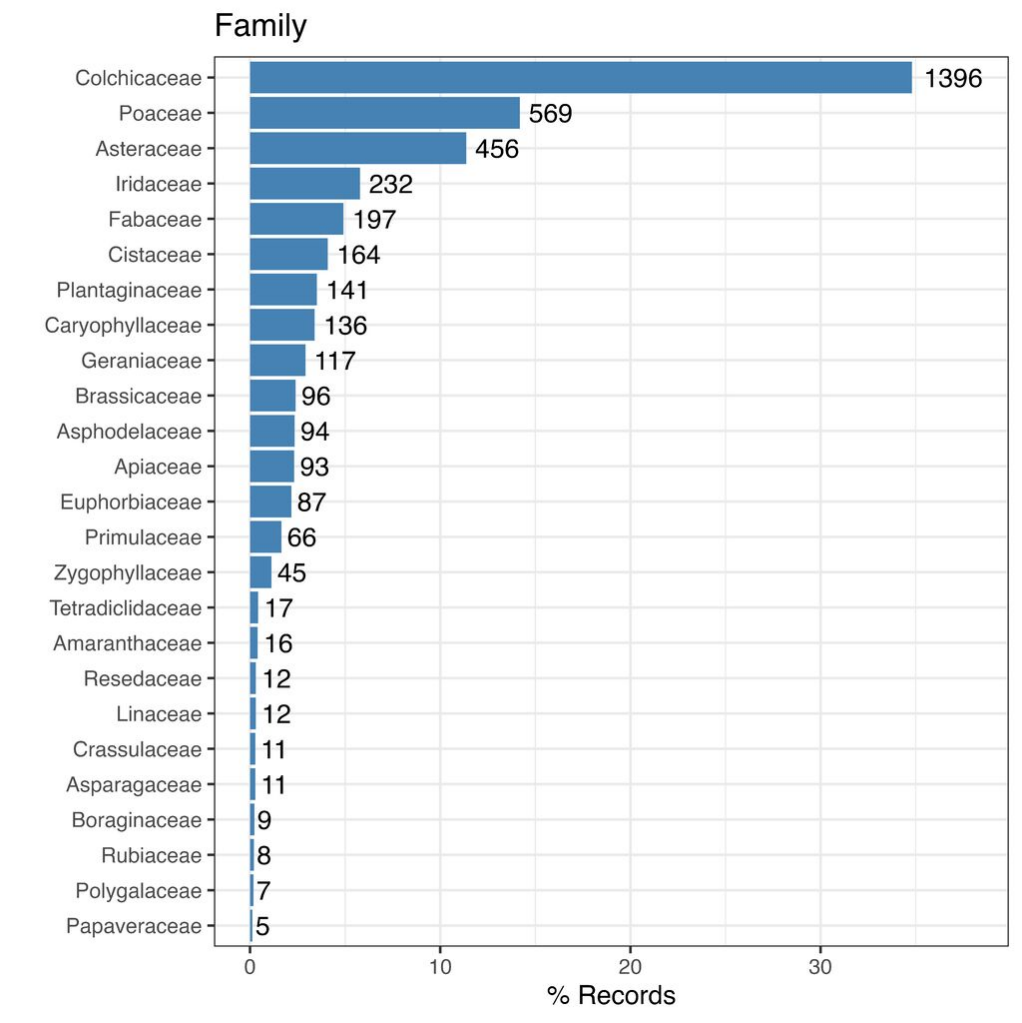
Taxonomic coverage (families). Percentage of dataset records by families. The numbers indicate the records of each family.

**Figure 4.**
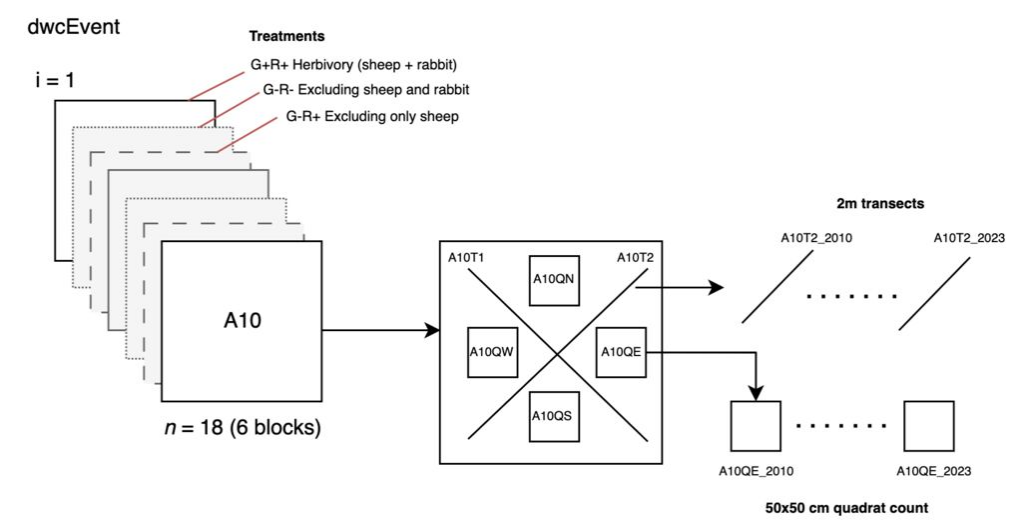
Scheme of the Event dataset. There are six blocks with three treatments per block (one plot by treatment): ***G+C***+ with herbivory by sheep and rabbits; ***G-C***+ excluding only sheep; and ***G-C***- excluding rabbits and sheep. In each of the 18 plots, there are two 2-m transects and four quadrats which were yearly monitored.

There are 100 taxa included in the dataset. The five most represented taxa in the dataset are: *Androcymbiumeuropaeum* (Lange) K. Richt. (34.74%), *Stipellulacapensis* (Thunb.) Röser & Hamasha (11.09%), *Gynandririssisyrinchium* (L.) Parl. (5.64%), *Filagopyramidata* L. (3.51%) and *Erodiumchium* (Burm.fil.) Willd. (2.37%). There are two classes represented in the dataset: Liliopsida (57.14%) and Magnoliopsida (42.86%). Twenty-one orders represented in the dataset being the five most represented: Liliales (34.74%), Poales (14.23%), Asterales (11.2%), Asparagales (8.14%) and Fabales (4.97%). There are 31 families included in the dataset (Fig. 3). The five families most represented in the dataset are: Colchicaceae (34.74%), Poaceae (14.23%), Asteraceae (11.2%), Iridaceae (5.64%) and Fabaceae (4.87%). Seventy-two genera are included in the dataset, with the five most represented being: *Androcymbium* (34.74%), *Stipellula* (11.09%), *Gynandriris* (5.64%), *Helianthemum* (4.28%) and *Filago* (3.83%).

## Temporal coverage

### Notes

2010-2023

## Collection data

### Collection name

This dataset belongs to the databases for monitoring Mediterranean silvopastoral systems ("*Bases de datos de seguimiento de sistemas silvopastorales mediterráneos*") (https://www.gbif.es/coleccion/bases-de-datos-de-seguimiento-de-sistemas-silvopastorales-mediterraneos/) generated and managed by the Service for the Assessment, Restoration and Protection of Mediterranean Agrosystems (SERPAM, *Servicio de Evaluación, Restauración y Protección de Agrosistemas Mediterráneos*) of the Estación Experimental del Zaidín (https://www.eez.csic.es/), belonging to Spanish National Research Council (CSIC).

## Usage licence

### Usage licence

Creative Commons Public Domain Waiver (CC-Zero)

### IP rights notes

This work is licensed under a Creative Commons Attribution Non Commercial (CC-BY-NC 4.0) Licence.

## Data resources

### Data package title

Monitoring data on the effect of domestic livestock and rabbits on *Androcymbiumeuropaeum* pastures

### Resource link

 https://doi.org/10.15470/jpjhuu

### Alternative identifiers

https://www.gbif.org/dataset/340aff40-1745-4d49-bf2a-adb2899bc428; https://ipt.gbif.es/resource?r=amoladeras

### Number of data sets

1

### Data set 1.

#### Data set name

Monitoring data on the effect of domestic livestock and rabbits on *Androcymbiumeuropaeum* pastures. Event

#### Data format

Darwin Core

#### Data format version

2.3

#### Description

Dataset about the annual monitoring of the effect of herbivorism on the conservation status of endangered species *Androcymbiumeuropaeum*. Since 2010, the SERPAM Department (Service for Evaluation, Restoration and Protection of Mediterranean Agrosystems) of the Zaidin Experimental Station belonging to the Spanish National Research Council (CSIC-EEZ), has been carrying out annual sampling to evaluate the effect of domestic and wild livestock (e.g. rabbits) on the pastures inhabited by *Androcymbiumeuropaeum*. A randomised block design with three treatments (type of management: rabbit and domestic herbivorism; only excluded to livestock; and excluded to rabbit and livestock) was performed. In each treatment, two types of monitoring were carried out: abundance estimation of *A.europaeum* by counting individuals on 50 cm x 50 cm quadrats; and plant species diversity in 2-m long transects using the modified Point-Quadrat method. This study was carried out in the Amoladeras Nature Reserve (Almería) within the Cabo de Gata-Níjar Natural Park (southern Spain). The dataset describes information from 2010 to 2023. Monitoring is performed annually. The dataset is deposited at GBIF (Pérez-Luque et al. 2023).

Our dataset is composed by three files: event, occurrence and extendedmeasurementorfact, with a total of 41 columns.

**Data set 1.** 

Column label	Column description
eventID	An identifier for the set of information associated with an Event (something that occurs at a place and time). May be a global unique identifier or an identifier specific to the dataset. http://rs.tdwg.org/dwc/terms/eventID.
parentEventID	An identifier for the broader Event that groups this and potentially other Events. http://rs.tdwg.org/dwc/terms/parentEventID.
samplingProtocol	The names of, references to, or descriptions of the methods or protocols used during a Event. http://rs.tdwg.org/dwc/terms/samplingProtocol.
sampleSizeValue	A numeric value for a measurement of the size (time duration, length, area or volume) of a sample in a sampling Event. http://rs.tdwg.org/dwc/terms/sampleSizeValue.
sampleSizeUnit	The unit of measurement of the size (time duration, length, area or volume) of a sample in a sampling event. http://rs.tdwg.org/dwc/terms/sampleSizeUnit.
eventDate	The date-time or interval during which an Event occurred. For occurrences, this is the date-time when the event was recorded. Not suitable for a time in a geological context. http://rs.tdwg.org/dwc/terms/eventDate.
fieldNumber	An identifier given to the event in the field. Often serves as a link between field notes and the Event. We used to identify the block and the treatment. http://rs.tdwg.org/dwc/iri/fieldNumber.
fieldNotes	One of a) an indicator of the existence of, b) a reference to (publication, URI), or c) the text of notes taken in the field about the event. We used to include the treatment. http://rs.tdwg.org/dwc/iri/fieldNotes.
countryCode	The standard code for the country in which the Location occurs. http://rs.tdwg.org/dwc/terms/countryCode.
municipality	The full, unabbreviated name of the next smaller administrative region than county (city, municipality etc.) in which the Location occurs. Do not use this term for a nearby named place that does not contain the actual Location. http://rs.tdwg.org/dwc/terms/municipality.
footprintWKT	A Well-Known Text (WKT) representation of the shape (footprint, geometry) that defines the Location. A Location may have both a point-radius representation and a footprint representation and they may differ from each other. http://rs.tdwg.org/dwc/terms/footprintWKT.
footprintSRS	The ellipsoid, geodetic datum or spatial reference system (SRS) upon which the geometry given in footprintWKT is based. http://rs.tdwg.org/dwc/terms/footprintSRS.
language	A language of the resource. http://purl.org/dc/terms/language.
institutionCode	The name (or acronym) in use by the institution having custody of the object(s) or information referred to in the record. http://rs.tdwg.org/dwc/terms/institutionCode.
collectionCode	The name, acronym, coden or initialism identifying the collection or dataset from which the record was derived. http://rs.tdwg.org/dwc/terms/collectionCode.
datasetName	The name identifying the dataset from which the record was derived. http://rs.tdwg.org/dwc/terms/datasetName.
ownerInstitutionCode	The name (or acronym) in use by the institution having ownership of the object(s) or information referred to in the record. http://rs.tdwg.org/dwc/terms/ownerInstitutionCode.
basisOfRecord	The specific nature of the data record. http://rs.tdwg.org/dwc/terms/basisOfRecord.
occurrenceID	An identifier for the Occurrence (as opposed to a particular digital record of the Occurrence). In the absence of a persistent global unique identifier, construct one from a combination of identifiers in the record that will most closely make the occurrenceID globally unique. http://rs.tdwg.org/dwc/terms/occurrenceID.
scientificName	The full scientific name, with authorship and date information, if known. When forming part of a Identification, this should be the name in lowest level taxonomic rank that can be determined. This term should not contain identification qualifications, which should, instead, be supplied in the identificationQualifier term. http://rs.tdwg.org/dwc/terms/scientificName
kingdom	The full scientific name of the kingdom in which the Taxon is classified. http://rs.tdwg.org/dwc/terms/kingdom.
phylum	The full scientific name of the phylum or division in which the Taxon is classified. http://rs.tdwg.org/dwc/terms/phylum.
class	The full scientific name of the class in which the Taxon is classified. http://rs.tdwg.org/dwc/terms/class.
order	The full scientific name of the order in which the Taxon is classified. http://rs.tdwg.org/dwc/terms/order.
family	The full scientific name of the family in which the Taxon is classified. http://rs.tdwg.org/dwc/terms/family.
genus	The full scientific name of the genus in which the Taxon is classified. http://rs.tdwg.org/dwc/terms/genus.
measurementID	An identifier for the MeasurementOrFact (information pertaining to measurements, facts, characteristics or assertions). May be a global unique identifier or an identifier specific to the dataset. http://rs.tdwg.org/dwc/terms/measurementID.
measurementType	The nature of the measurement, fact, characteristic or assertion. http://rs.tdwg.org/dwc/terms/measurementType.
measurementValue	The value of the measurement, fact, characteristic or assertion. http://rs.tdwg.org/dwc/terms/measurementValue.
measurementUnit	The units associated with the measurementValue. http://rs.tdwg.org/dwc/terms/measurementUnit.
measurementDeterminedDate	The date on which the MeasurementOrFact was made. http://rs.tdwg.org/dwc/terms/measurementDeterminedDate.
measurementMethod	A description of or reference to (publication, URI) the method or protocol used to determine the measurement, fact, characteristic or assertion. http://rs.tdwg.org/dwc/terms/measurementMethod.
measurementRemarks	Comments or notes accompanying the MeasurementOrFact. http://rs.tdwg.org/dwc/terms/measurementRemark.
occurrenceStatus	A statement about the presence or absence of a Taxon at a Location. http://rs.tdwg.org/dwc/terms/occurrenceStatus.
decimalLatitude	The geographic latitude (in decimal degrees, using the spatial reference system given in geodeticDatum) of the geographic centre of a Location. http://rs.tdwg.org/dwc/terms/decimalLatitude.
decimalLongitude	The geographic longitude (in decimal degrees, using the spatial reference system given in geodeticDatum) of the geographic centre of a Location. http://rs.tdwg.org/dwc/terms/decimalLongitude.
geodeticDatum	The ellipsoid, geodetic datum or spatial reference system (SRS) upon which the geographic coordinates given in decimalLatitude and decimalLongitude are based. http://rs.tdwg.org/dwc/terms/geodeticDatu.
genericName	The genus part of the scientificName without authorship. http://rs.tdwg.org/dwc/terms/genericName.
specificEpithet	The name of the first or species epithet of the scientificName. http://rs.tdwg.org/dwc/terms/specificEpithet.
taxonRank	The taxonomic rank of the most specific name in the scientificName. http://rs.tdwg.org/dwc/terms/taxonRank.
scientificNameAuthorship	The authorship information for the scientificName formatted according to the conventions of the applicable nomenclaturalCode. http://rs.tdwg.org/dwc/terms/scientificNameAuthorship.

## Additional information

If you use the data, please cite as: Pérez-Luque A J, Ramos-Font M E, Tognetti M J, Montoya Román C, Tribaldos Anda C, Robles A B (2023). Monitoring data on the effect of domestic livestock and rabbits on *Androcymbiumeuropaeum* pastures. Version 2.3. Estación Experimental del Zaidín (CSIC). Sampling event dataset https://doi.org/10.15470/jpjhuu accessed via GBIF.org on 09-10-2023.
